# P-2227. Real-Life Utilization and Clinical Impact of Karius Testing in Solid Organ Transplant Recipients: A Retrospective Analysis

**DOI:** 10.1093/ofid/ofae631.2381

**Published:** 2025-01-29

**Authors:** Jesal R Shah, M Rizwan Sohail, Mayar Al Mohajer, Sarwat Khalil

**Affiliations:** Baylor College of Medicine, Houston, Texas; Baylor College of Medicine, Houston, Texas; Baylor College of Medicine, Houston, Texas; Baylor College of Medicine, Houston, Texas

## Abstract

**Background:**

Metagenomic next-generation sequencing such as Karius test (KT), is increasingly being used for microbial detection in various clinical syndromes, including sepsis, Fever of Unknown Origin (FUO), pneumonia, and cardiovascular infection. However, data regarding the utility of KT in solid organ transplant recipients (SOTR) are lacking. There is a need to assess its clinical impact and appropriate use considering its cost, potential for detection of commensal organisms, and lack of antimicrobial susceptibility data. Our study focuses on the real-world clinical impact of KT in SOTR.

**Figure:**
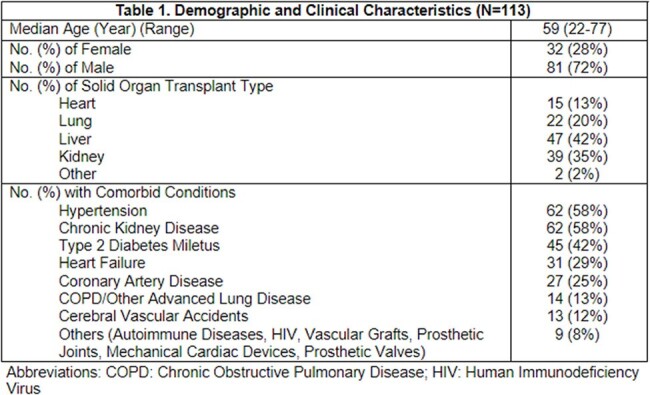

**Methods:**

We performed a retrospective review of all adult SOTR who underwent KT at Baylor St Luke’s Medical Center from March 2017 to February 2023. Clinical impact (positive, neutral, and negative) was assessed using standardized objective criteria utilized in prior studies. Descriptive analysis with patient and clinical characteristics was completed.

**Figure:**
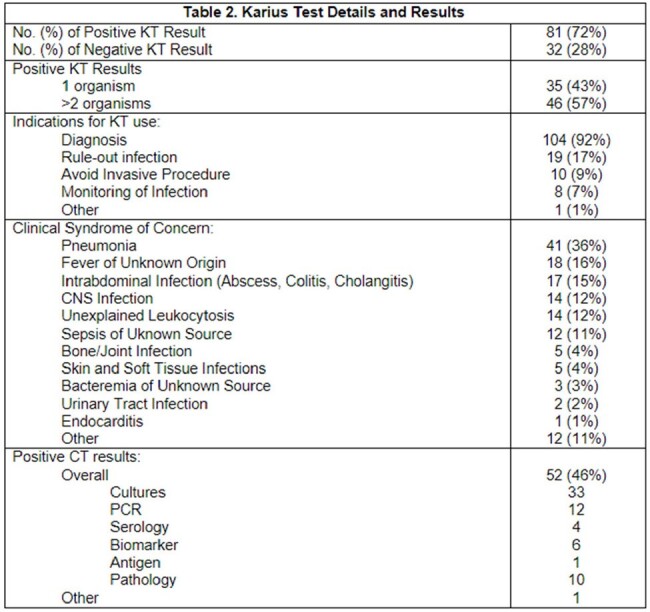

**Results:**

A total of 113 KT in liver (42%), kidney (35%), lung (20%) and heart (13%) transplant recipients were completed in the study period (Table 1). The most common clinical syndromes where KT was ordered included: pneumonia (36%), FUO (16%), and intrabdominal infections (15%). Majority (81, 72%) of the KT results were positive for microbiological organisms (Table 2). Overall, 52 (46%) of conventional microbiological tests (CT) were also positive. Ultimately, 27 (24%) of cases were identified with positive clinical impact from KT testing, 87 (77%) neutral, and 4 (4%) negative, respectively (Table 3).

**Figure:**
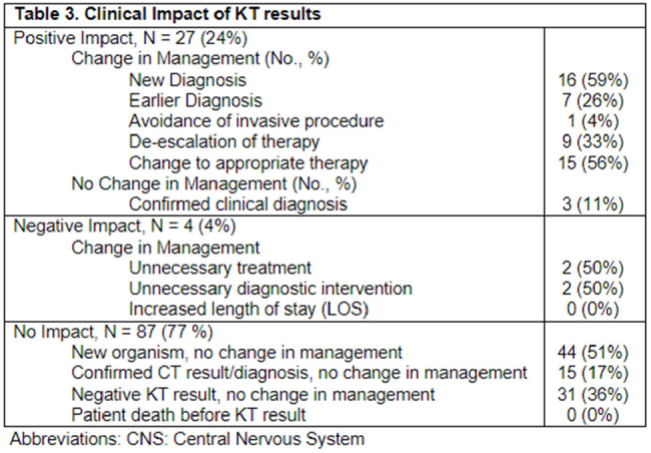

**Conclusion:**

In SOTR, KT can add a positive clinical impact and identify microorganisms beyond conventional microbiological testing across clinical syndromes. However, larger prospective studies are needed to define the optimal timing and utilization in diagnostic evaluation algorithms for syndrome-specific work-up in SOTR.

**Disclosures:**

M. Rizwan Sohail, MD, Medtronic: Advisor/Consultant|Medtronic: Honoraria|Philips: Advisor/Consultant|Philips: Honoraria

